# Chronic Myeloid Leukemia Masked by Massive Splenomegaly: Splenectomy as a Strategy in a Resource-Limited Setting

**DOI:** 10.7759/cureus.109341

**Published:** 2026-05-21

**Authors:** Sela Bun

**Affiliations:** 1 Surgery, Techo Santepheap National Hospital, Phnom Penh, KHM

**Keywords:** bcr-abl1, chronic myeloid leukemia, diagnostic delay, leukocytosis, myeloproliferative neoplasm, resource-limited setting, splenectomy, splenomegaly

## Abstract

Massive splenomegaly may obscure the diagnosis of underlying hematologic malignancies, particularly in resource-limited settings. Chronic myeloid leukemia (CML) typically presents with marked leukocytosis; however, atypical presentations may lead to diagnostic delay and inappropriate initial management.

A 40-year-old male presented with progressive splenomegaly and abdominal fullness. Preoperative laboratory evaluation demonstrated leukocytosis with associated mild anemia. Imaging revealed massive hepatosplenomegaly, and a provisional diagnosis of Banti syndrome was made. The patient underwent open splenectomy for symptomatic massive splenomegaly. Histopathological examination of the resected spleen was non-diagnostic. Postoperatively, persistent, progressive leukocytosis and thrombocytosis prompted further hematologic evaluation. Bone marrow examination suggested a myeloproliferative neoplasm. Due to limited access to molecular diagnostics, confirmatory testing was delayed. Subsequent real-time polymerase chain reaction (PCR) confirmed BCR-ABL1 positivity, establishing the diagnosis of classical CML. The patient was treated with cytoreductive therapy, followed by imatinib, with a favorable clinical and hematologic response.

This case highlights the potential for massive splenomegaly to mask CML and emphasizes the importance of early molecular testing. Splenectomy may unmask underlying hematologic malignancy through significant postoperative hematologic changes.

## Introduction

Massive splenomegaly is a clinically significant finding with a broad differential diagnosis, including infectious diseases, hematologic malignancies, and portal hypertensive disorders. In resource-limited settings, diagnostic evaluation may be constrained by limited access to advanced molecular testing, increasing the risk of delayed or inaccurate diagnosis.

Chronic myeloid leukemia (CML) is a myeloproliferative neoplasm characterized by the presence of the BCR-ABL1 fusion gene resulting from a reciprocal translocation between chromosomes 9 and 22 [1]. Splenomegaly is a common clinical feature of CML; however, the degree of enlargement varies and may occasionally dominate the clinical presentation, potentially obscuring the underlying diagnosis [2].

The introduction of tyrosine kinase inhibitors has transformed the management of CML, rendering splenectomy rarely indicated except in selected cases [3]. Nevertheless, in settings where diagnostic resources are limited, patients with symptomatic splenomegaly may undergo surgical intervention prior to a definitive diagnosis.

We report a case of delayed diagnosis of BCR-ABL1-positive CML presenting as massive splenomegaly, initially managed with splenectomy, which subsequently revealed a marked postoperative hematologic surge and diagnostic challenge.

## Case presentation

A 40-year-old male farmer presented with progressive abdominal distension and early satiety. He had been aware of splenomegaly since 2020, which had gradually increased in size. There was no history of fever, night sweats, or significant weight loss.

On physical examination, a firm, non-tender, massively enlarged spleen was palpable, extending into the left iliac fossa and crossing the midline, approximately 15-20 cm below the left costal margin.

Preoperative laboratory investigations demonstrated leukocytosis (approximately 38 × 10⁹/L) with a marked left shift, including the presence of myelocytes and metamyelocytes, associated with microcytic anemia. Laboratory investigations at presentation and during follow-up are summarized in Table 1.

**Table 1:** Laboratory findings over the clinical course. WBC: white blood cell count; LDH: lactate dehydrogenase

Parameter	Normal Range	December 2024	March 2025 (Pre-op)	March 2025 (Post-op)	September 2025 (Peak)	March 2026 (Follow-up)
WBC (×10⁹/L)	4-10	33.2	37.5	70.3	161.3	9.8
Hemoglobin (g/dL)	13-17	9.6	10.5	10.1	17.4	16.5
Platelets (×10⁹/L)	150-450	409	467	880	1031	436
Neutrophils (×10⁹/L)	2-7	15.3	28.1	42.9	53	6.2
Myelocytes (%)	0	16	6	15	-	-
Metamyelocytes (%)	0	10	4	10	-	-
LDH (U/L)	132-248	833	-	-	-	-

Contrast-enhanced computed tomography revealed hepatosplenomegaly, with the spleen measuring approximately 287 × 116 mm and associated portal vein dilation, without focal lesions. Based on the clinical and radiologic findings, a provisional diagnosis of Banti syndrome was made (Figure 1).

**Figure 1:**
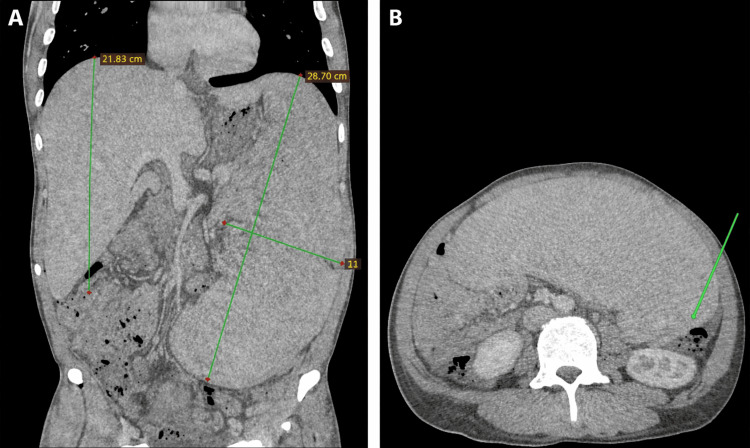
Contrast-enhanced computed tomography demonstrating massive splenomegaly. (A) Coronal view showing a massively enlarged spleen occupying a large portion of the abdominal cavity, with a maximal craniocaudal length of approximately 28.7 cm. (B) Axial view illustrating the extent of splenic enlargement and displacement of adjacent abdominal structures.

The patient underwent elective open splenectomy on March 25, 2025. Intraoperatively, the spleen occupied more than 50% of the abdominal cavity. No lymphadenopathy or other intra-abdominal abnormalities were identified (Figure 2).

**Figure 2:**
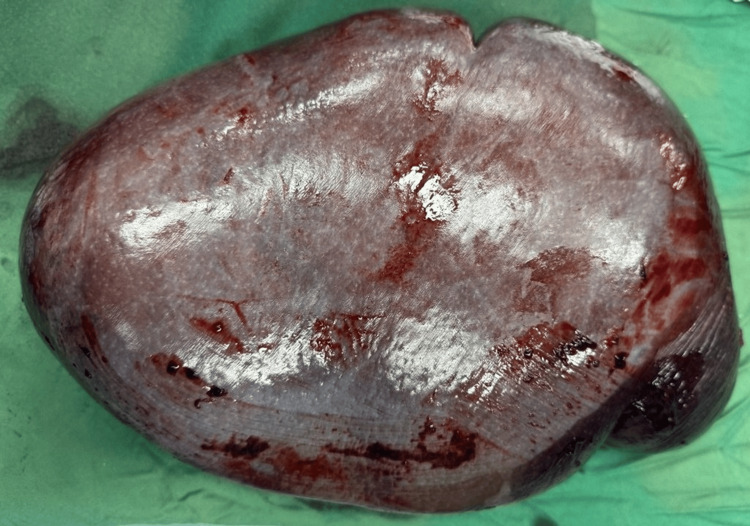
Gross specimen of resected spleen demonstrating massive splenomegaly. Gross specimen of the resected spleen demonstrating marked enlargement consistent with massive splenomegaly. The external surface appears smooth, with a congestive appearance. The specimen measured approximately 30 × 12 × 4 cm.

Histopathological examination of the resected spleen revealed features of congestive splenomegaly, characterized by marked congestion of the red pulp sinusoids and relative atrophy of the white pulp, with preserved splenic architecture. There was no evidence of malignancy or significant extramedullary hematopoiesis (Figure 3).

**Figure 3:**
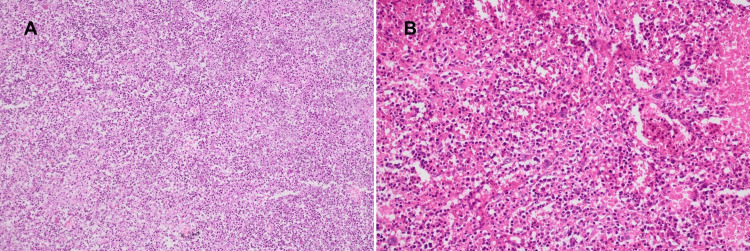
Histopathological features of congestive splenomegaly in the resected spleen. (A) Low-power view demonstrating diffuse congestion of the red pulp with relative atrophy of the white pulp (hematoxylin and eosin stain). (B) High-power view showing dilated sinusoids filled with red blood cells and mixed inflammatory cells, without evidence of malignant infiltration.

Postoperatively, the patient developed marked leukocytosis and thrombocytosis. The white blood cell count increased from approximately 40 × 10⁹/L preoperatively to over 70 × 10⁹/L immediately after surgery, reaching 161 × 10⁹/L within six months. Platelet counts similarly rose to greater than 1000 × 10⁹/L.

Bone marrow examination performed at the end of September 2025 revealed hypercellularity with a myeloid-to-erythroid ratio of approximately 7:1, granulocytic proliferation with left shift, and basophilia, suggestive of a myeloproliferative neoplasm. No formal bone marrow fibrosis grading was available, as the evaluation was based primarily on bone marrow aspirate/myelogram findings. In the absence of molecular confirmation at that time, atypical CML was included in the initial differential diagnosis.

Due to limited local availability of molecular testing, confirmatory analysis was delayed. Real-time polymerase chain reaction (PCR) performed in January 2026 subsequently confirmed the presence of the BCR-ABL1 fusion gene (b2a2 transcript), establishing the diagnosis of CML.

The patient was initially managed with hydroxyurea for cytoreduction and allopurinol for tumor lysis prophylaxis. Following confirmation of the diagnosis, targeted therapy with imatinib (400 mg daily) was initiated.

At follow-up, the patient demonstrated normalization of leukocyte counts and remained clinically stable, with good tolerance to treatment (Figure 4).

**Figure 4:**
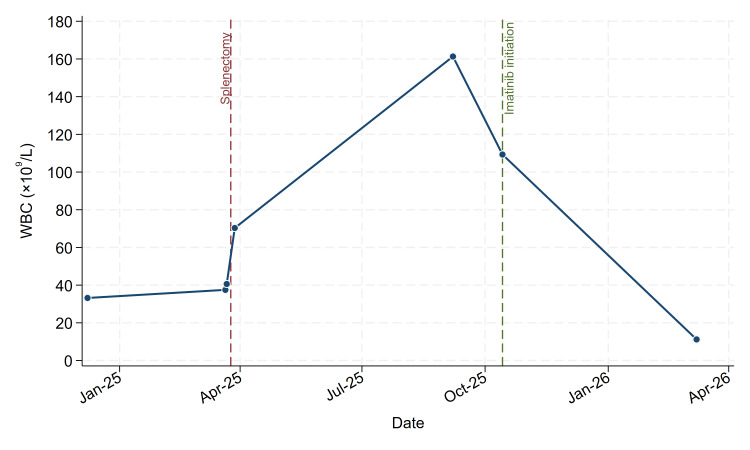
Evolution of WBC count over time, demonstrating a marked increase following splenectomy and subsequent normalization after initiation of imatinib therapy. The red dashed line indicates the date of splenectomy, and the green dashed line indicates initiation of imatinib therapy. Values were obtained from peripheral blood measurements during follow-up. WBC: white blood cell count

## Discussion

This case highlights the diagnostic challenge of CML presenting as massive splenomegaly, particularly in resource-limited settings.

Although the patient already had preoperative hematologic abnormalities suggestive of a myeloproliferative neoplasm, including leukocytosis, neutrophilia, thrombocytosis, and a left shift with myelocytes and metamyelocytes, the clinical pathway was initially dominated by longstanding symptomatic massive splenomegaly and radiologic features suggestive of portal hypertensive disease. Although splenomegaly is a common feature of CML and other myeloproliferative neoplasms, it may occasionally dominate the clinical picture and obscure the underlying hematologic disorder. In some patients, splenic enlargement precedes or outweighs laboratory abnormalities, leading to diagnostic uncertainty [3]. In the present case, progressive splenic enlargement over several years, in association with hepatomegaly and imaging findings suggestive of portal venous involvement, initially favored a diagnosis of Banti syndrome. Although leukocytosis and a left shift were present, these findings were interpreted as nonspecific in the context of massive splenomegaly, while anemia was attributed to hypersplenism. In addition, imaging findings and histopathological examination demonstrating congestive splenomegaly without evidence of malignancy further supported an alternative diagnosis, such as a portal hypertensive or vascular etiology. These findings contributed to the initial misclassification and delayed recognition of the underlying disease.

Following splenectomy, the patient demonstrated persistent and progressive leukocytosis and thrombocytosis during follow-up, which prompted further hematologic evaluation, including bone marrow examination. In retrospect, earlier bone marrow assessment could have been considered given the preoperative leukocytosis and left shift; however, the initial clinical pathway was largely driven by longstanding symptomatic massive splenomegaly and radiologic concern for portal hypertensive disease.

The spleen serves as a major site of sequestration and turnover of blood cells. Its removal can result in a significant increase in circulating leukocytes and platelets, a phenomenon that has been described in patients with myeloproliferative neoplasms undergoing splenectomy [4,5]. In the present case, the marked postoperative leukocytosis and thrombocytosis represented an important clinical turning point that prompted reconsideration of the diagnosis and further hematologic evaluation. Although hematologic abnormalities suggestive of an underlying myeloproliferative neoplasm were already present preoperatively, splenectomy made these abnormalities more evident during postoperative follow-up.

This case underscores the limitations of diagnostic evaluation in the absence of timely hematologic assessment and molecular testing. While bone marrow findings may suggest a myeloproliferative neoplasm, definitive diagnosis of CML requires demonstration of the BCR-ABL1 fusion gene [6,7]. In this case, initial uncertainty - including consideration of atypical CML - reflected reliance on morphology alone. In retrospect, earlier hematologic evaluation, including bone marrow examination, might have provided important diagnostic clues suggestive of an underlying myeloproliferative neoplasm even before molecular confirmation and could potentially have avoided splenectomy. The subsequent confirmation of BCR-ABL positivity by real-time PCR established the diagnosis of classical CML and allowed initiation of targeted therapy in accordance with current guidelines [7].

In many low- and middle-income settings, access to advanced molecular diagnostics is limited or delayed. As a result, clinicians may rely more heavily on clinical, radiologic, and histopathologic findings, which can contribute to delayed hematologic diagnosis and potentially avoidable surgical intervention. Similar diagnostic challenges have been reported in resource-constrained environments, where gaps in laboratory capacity affect timely and accurate diagnosis of hematologic malignancies [8].

Comparison with previously reported cases further emphasizes the distinct diagnostic sequence observed in this patient. While splenectomy was historically described as a therapeutic option in patients with established CML and symptomatic massive splenomegaly [9], most reported cases involved a confirmed diagnosis prior to surgical intervention. In contrast, in the present case, longstanding massive splenomegaly initially dominated the clinical presentation, and subsequent postoperative hematologic progression prompted further hematologic evaluation that ultimately established the diagnosis of CML.

To our knowledge, such presentations remain uncommon, with splenectomy preceding the diagnosis of CML and postoperative leukocytosis serving as a key diagnostic clue prompting further hematologic evaluation.

Taken together, massive splenomegaly should prompt careful evaluation for an underlying hematologic malignancy. Early consideration of a hematologic diagnosis and appropriate investigations, including peripheral blood evaluation and bone marrow assessment, is essential to avoid potentially unnecessary invasive procedures such as splenectomy. This is particularly important in resource-limited settings, where diagnostic delays remain common and may adversely affect patient outcomes.

## Conclusions

Massive splenomegaly may obscure the diagnosis of CML, particularly when a hematologic diagnosis is not initially considered. Splenectomy may make the hematologic abnormalities more evident through significant postoperative hematologic changes. This case highlights the importance of considering CML in the differential diagnosis of unexplained splenomegaly and underscores the need for early bone marrow assessment and molecular studies whenever feasible to avoid potentially unnecessary surgical intervention and ensure timely initiation of appropriate targeted therapy.
